# Evaluation of HIV-1 rapid diagnostic tests in the context of viral genetic diversity in Libreville (Gabon)

**DOI:** 10.11604/pamj.2022.42.194.35035

**Published:** 2022-07-11

**Authors:** Yann Vital Sima Biyang, Soulemane Parkouda, Berthold Bivigou-Mboumba, Berthe Amélie Iroungou, Augustin Mouinga Ondeme, Cyrille Bisseye

**Affiliations:** 1 Laboratoire de Biologie Moléculaire et Cellulaire (LABMC), Université des Sciences et Techniques de Masuku, BP 943, Franceville, Gabon,; 2 Centre Hospitalier Régional Georges RAWIRI (CHRGR), Lambaréné, Gabon,; 3 Unité Mixte de Recherches sur le VIH et les Maladies Infectieuses Associées (UMR, MIA), Hôpital d´Instruction des Armées Omar BONGO ONDIMBA, Libreville, Gabon,; 4 Centre Interdisciplinaire de Recherches Médicales de Franceville (CIRMF), Franceville, Gabon

**Keywords:** HIV-1, genotypes, rapid detection test, qRT-PCR, sensitivity, specificity

## Abstract

**Introduction:**

in order to promote rapid care of HIV-positive people and to reduce the human immunodeficiency virus (HIV) transmission in Gabon, the national screening algorithm is essentially based on rapid diagnostic tests (RDTs). However, most of these RDTs are not evaluated. Their sensitivities and specificities remain unknown locally. The aim of this study was to determine the diagnostic performance of 3 RDTs used for HIV-1> screening in Gabon.

**Methods:**

of the one hundred and sixteen (116) samples tested, 60 plasmas were HIV-1 positive with known genotypes and viral loads; 51 sera were HIV-1 negative while 5 had an undetermined serological status. All the samples were tested by quantitative RT-PCR (Gold standard) and by the following RDTs: Vikia, Alere Combo and Alere Determine. The sensitivities and specificities of the different RDTs were calculated using Epi Info version 6.04dfr. The level of agreement between tests was determined by Cohen´s Kappa test.

**Results:**

the three RDTs´ sensitivity according to HIV-1/M subtypes was 100% (95% CI: 92.6-100) while their specificities ranged from 94.6% (95% CI: 84.2-98.6) for the Vikia test to 96.4% (95% CI: 86.6-99.4) for the Alere Combo and Alere Determine tests, respectively. The concordances between the three RDTs were excellent with kappa values ranging from 0.931 (95% CI: 0.864-0.977) to 0.948 (95% CI: 0.890-1.00).

**Conclusion:**

the three RDTs showed a maximum sensitivity of 100% and specificities ranging from 94.6% to 96.4%. The specificities obtained with these RDTs are lower than those recommended by the WHO for their inclusion in an HIV-1 screening algorithm.

## Introduction

Human immunodeficiency virus (HIV) infection remains a public health problem in Gabon. Its seroprevalence was estimated at 4.1% in 2012 during the last demographic and health survey carried out in the country [1]. In Gabon, in order to promote the rapid treatment of HIV-seropositive patients and to reduce the transmission of HIV, the diagnosis of this virus is based essentially on Rapid Diagnostic Tests (RDTs) in the absence of 4th generation immunoenzymatic tests and nucleic acid tests (NAT) by PCR in health facilities because of their prohibitive cost. However, in order to be eligible for an HIV screening algorithm according to the recommendations of the World Health Organization (WHO), the RDTs must have a sensitivity ≥ 99% and a specificity ≥ 98% [2].

In Gabon, the national HIV screening algorithm offers three RDTs: Alere Combo Ag/Ab 4th generation HIV-1/2, Alere Determine HIV-1/2 and SD HIV-1/2 3.0 (Multi). HIV infection in this country is characterized by a high viral genetic diversity which could lead to the absence of detection of certain HIV-1 subtypes for RDTs. Indeed, HIV-1 is divided into four groups: M (Major), O (Outlier), N (non-M, non-O), and P [3]. Among the HIV-1 groups, group M is responsible for the pandemic of HIV infection [4]. This M group includes ten subtypes (A-D, F-H, and J-L) with several Circulating Recombinant Forms (CRFs) (Yamaguchi *et al*., 2020) [5]. In Gabon, the HIV group M is the main circulating strain [6] with several subtypes and recombinant forms identified [7-9] while group O is poorly represented with a prevalence of 0.9% [10]. The available RDTs are often introduced on the local market without prior evaluation and could have the disadvantage of not being adapted to the viral subtypes circulating in the country. An evaluation before their use on the local market and the regular evaluation of their effectiveness is therefore necessary. Some HIV RDTs have already been evaluated in previous studies. The reported sensitivities and specificities were 99.60% and 100% for INSTI HIV1/HIV2 test [11] and 90.9% and 100% for Alere Determine HIV1/HIV2 test [12]. The diversity of HIV-1 in Gabon could be constantly changing due to the geographical proximity with Cameroon, a country known to harbor the majority of HIV-1 subtypes [13,14]. This study was carried out to evaluate three RDTs used in HIV screening in Libreville, Gabon in a context of viral genetic diversity.

## Methods

**Study design and settings:** this was a prospective cross-sectional study that took place from August to November 2020 at the *Unité Mixte de Recherches sur le VIH et les Maladies Infectieuses Associées (UMR, MIA)* and the Centre Interdisciplinaire de Recherches Médicales de Franceville (CIRMF), located at the *Hôpital des Instructions des Armées Omar Bongo Ondimba (HIAOBO)*, based in Libreville, the capital of Gabon.

**Participants:** the study concerned samples from HIV-positive patients in consultation for a viral load and the identification of antiretroviral resistance mutations; and on sera from patients with negative or indeterminate HIV serology. A total of 60 group-M HIV-1+ plasmas were tested. The genotypic proportions of HIV-1 were respectively 46.7% (28/60) for CRF02-AG; 18.3% (11/60) for A; 11.7% (7/60) for G; 5% (3/60) for A/G; 5% (3/60) for CRF06-Cpx; 3.3% (2/60) for CRF04-Cpx; 3.3% (2/60) for H; 1.7% (1/60) for B; 1.7% (1/60) for D; 1.7% (1/60) for A/J and 1.7% (1/60) for CRF18-Cpx. In addition, 51 negative sera and 5 with undetermined HIV serology were also tested.

**HIV diagnostic and confirmation:** the Alere Combo Ag/Ab 4th generation HIV1/2 (Alere Medical., Ltd Japan), Alere Determine HIV1/2 (Alere Medical., Ltd Japan) and Vikia HIV1/2 (Bio-Mérieux, France) RDTs were evaluated in this study. These tests based on the principle of immunochromatography for the qualitative detection of anti-HIV-1/2 antibodies were used according to the manufacturers´ recommendations. The performances stated by the manufacturers varied from 99.4% to 100% for the sensitivities and from 99.4% to 99.9% for the specificities. According to the manufacturer's instructions, the 5 undetermined patient sera were tested for HIV-1 group O using the Mini Vidas (Biomérieux, France) and not detectable by the GENERIC HIV Charge Viral kit (Biocentric, Bandol-France) and all the samples were tested by qRT-PCR, which served as a reference test for the validity of the RDTs.

**Figure 1:**
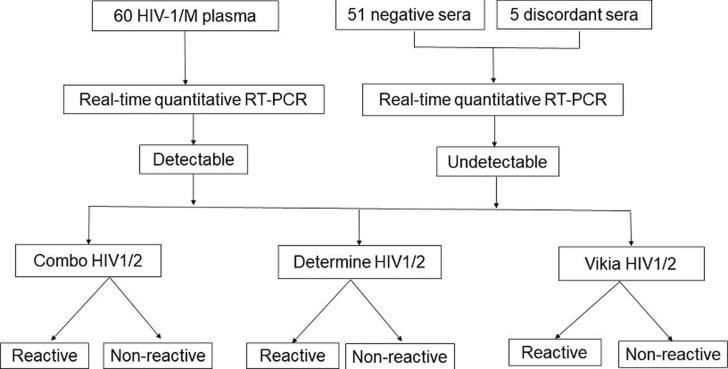
algorithm of HIV testing in the study

Viral RNA was extracted with the Arrow Viral RNA semi-automated extractor (DiaSorin, Ireland Ltd). Plasma viral load was quantified by real-time quantitative PCR. Briefly, 250 μL of serum was added to 5 microliters of proteinase K and 2 microliters of internal control for a final elution volume of 50 μL. The reverse transcription reaction was assessed using the GENERIC HIV Charge Viral Kit (Biocentric, Bandol, France) in a reaction volume of 20 μL (10 μL RNA+10 μl mix) with the Fluorocycler 96 Real-time PCR machine (Hain Lifescience) according to the following amplification program: 50°C for 10 minutes for reverse transcription; 95°C for 5 minutes of denaturation; followed by 50 cycles including denaturation at 95°C for 15 seconds and annealing at 60°C for 1 min. The HIV detection algorithm used during the study is shown in Figure 1.

**Statistical analysis:** data were entered using Microsoft Excel version 2016. R software (version R x 64.3.6.2) was used for the sensitivities, specificities, positive predictive values (PPV) and negative predictive values (NPV) determination, with 95% confidence intervals. Cohen's Kappa coefficient (http://graphpad.com/quickcalcs/kappa/ ) was calculated to assess the level of agreement between the different RDTs for the detection of HIV.

**Ethical consideration:** this study was approved by the HIAOBO Ethical Committee. All adults ≥18 years of age gave informed consent before participating in the study.

## Results

**Sociodemographic characteristics of patients:** a total of 116 patients were included in this study. Of these samples, 51.7% (60/116) were HIV-positive while 44% (51/116) were HIV-negative. Five (5) patients with indeterminate HIV serology were also included. Women were mostly more represented than men (66.4% vs 33.6%) both in HIV-positive patients (37.9% vs 13.8%) and HIV-negative patients (26.7% vs. 17.3%). The patients´ age varied from 18 to 72 years with a mean of 40 years ± 12.

**Performance of RDTs in the diagnosis of HIV-1:** the 60 HIV-1 positive plasmas belonged to group M with 11 distinct subtypes. All viral subtypes were detected by the three RDTs (Table 1). Of the 60 HIV+ and 56 HIV- samples detected by qRT-PCR, the Determine HIV-1/2 and Combo HIV1/2 RDTs found 60 HIV+ and 54 HIV- samples, respectively. Only 2 negative samples by qRT-PCR were positive for both RDTs. The Vikia HIV1/2 test detected all 60 HIV+ samples while only 53/56 HIV- samples were determined (Table 2).

**Table 1:** RDTs’ reactivities according to HIV-1/M subtypes

HIV-1/M Subtypes	HIV Viral Load median Log10 copies/mL (Min-Max)	Combo		Determine		Vikia	
		Positive	Negative	Positive	Negative	Positive	Negative
A (n=11)	5.6 (3.8-7)	11	0	11	0	11	0
B (n=1)	4.05	1	0	1	0	1	0
D (n=1)	4.74	1	0	1	0	1	0
G (n=7)	5.85 (4.31-6.34)	7	0	7	0	7	0
H (n=2)	4.95 (4.88-5.01)	2	0	2	0	2	0
A/G (n=3)	5.08 (3.43-6.56)	3	0	3	0	3	0
A/J (n=1)	6.64	1	0	1	0	1	0
CRF02-AG (n=28)	4.76 (2.67-6.7)	28	0	28	0	28	0
CRF04-Cpx (n=2)	5.88 (5.09-6.15)	2	0	2	0	2	0
CRF06-Cpx (n=3)	4.99 (3.72-6.30)	3	0	3	0	3	0
CRF18-Cpx (n=1)	5.22	1	0	1	0	1	0

**Table 2:** comparison of RDTs and qRT-PCR for the detection of HIV

		qRT-PCR			Tests performance			
		Pos	Neg	Total	Sensitivity % (95% CI)	Specificity % (95% CI)	PPV % (95% CI)	NPV % (95% CI)
**Combo 4th Generation**	Pos	60	2	62	100 (92.5-100)	96.4 (86.6-99.4)	96.8 (87.8-99.4)	100 (91.7-100)
	Neg	0	54	54				
	Total	60	56	56				
**Determine Alere**	Pos	60	2	62	100 (92.5-100)	96.4 (86.6-99.4)	96.8 (87.8-99.4)	100 (91.7-100)
	Neg	0	54	54				
	Total	60	56	56				
**Vikia**	Pos	60	3	63	100 (92.5-100)	94,6 (84.9-98.3)	95.2 (86.6-98.5)	100 (91.7-100)
	Neg	0	53	53				
	Total	60	56	56				

Neg: Negative; Pos: Positive; PPV: Positive Predictive Values; NPV: Negative Predictive Values; CI: Confidence Interval

The sensitivity of the 3 RDTs compared to quantitative RT-PCR for the detection of HIV-1 M subtypes was 100% (95% CI = 92.5-99.9). The specificity of the Combo Ag/Ab 4^e^G and Determine tests was 96.4% (95% CI = 86.6-99.3) while their positive and negative predictive values were 96.7% (95% CI = 87.8-99.4) and 100% (95% CI = 91.7-100), respectively. The Vikia test showed a specificity of 94.6% (95% CI = 84.1-98.6), its positive and negative predictive values were 95.2% (95% CI = 85.8-98.7) and 100% (95% CI = 91.6-100), respectively (Table 2).

We also compared the levels of agreement between the different RDTs used for HIV detection. These agreements were excellent for HIV detection between Combo and Determine on one hand and between Combo/Vikia and Vikia/Determine on the other hand with kappa values of 0.931 (95% CI = 0.865-0.977) and 0.948 (95% CI = 0.891-1.00), respectively (Table 3).

**Table 3:** comparison of RDTs for the detection of HIV

RDTs	Po%	Pe%	Kappa	95% CI
**Combo/Determine**	96.55	50.24	0.931	0.864-0.977
**Combo/Vikia**	97.41	50.30	0.948	0.890-1.00
**Determine/Vikia**	97.41	50.30	0.948	0.890-1.00

Po = proportion of observed agreement; Pe = proportion of expected agreement; CI= Confidence interval

## Discussion

The wide use of RDTs in HIV screening in Gabon and the high viral genetic diversity observed implies regular evaluation of these tests performance for their rational use in the national screening algorithm. In this study, three HIV RDTs used in Gabon were evaluated on a panel of confirmed HIV-positive samples, negative and undetermined samples.

The sensitivities of the three RDTs for HIV-1 M subtypes detection were 100%. These sensitivities are similar to those reported in an earlier study carried out in Cameroon [15]. Our results are also similar to those reported in France for the Vikia and Alere Combo tests [16]. However, they are different from the results obtained for the Alere Determine test in Congo [17] and Gabon [12] with the sensitivities of 57.1% and 90.9%, respectively. The difference between the sensitivities observed could be partly explained by the type of samples tested. Indeed, in the present study, we tested samples from confirmed HIV-positive patients, while in the two studies carried out in Congo and Gabon, they were blood donors who may have been diagnosed during the seroconversion phase. With regard to the study performed in Thailand, the evaluation of the Alere Combo test has been done with samples from patients in the acute phase of the infection, a period during which RDTs show reduced sensitivities [18]. This difference could also be attributed to the genetic variability of HIV [15]. RDTs could present a risk both in routine diagnosis and for transfusion safety in geographical areas where the qualification of blood bags is done using them, such as sub-Saharan Africa, including Gabon. Indeed, a previous study had reported a high HIV prevalence of 3.1% among blood donors in rural areas [19]. This could constitute a threat to the transfusion safety of recipients in these regions. Indeed, financial and technical resources are necessary to set up a strict algorithm including tests capable of circumventing the HIV genetic variability. Furthermore, molecular amplification tests by PCR which could minimize the transmission of this virus by blood transfusion in Gabon are needed.

The RDTs´ specificities ranged from 94.6% for Vikia test to 96.4% for the Alere Determine and Combo tests. These specificities were closer to those found in Cameroon, which ranged from 95.8% to 97.1% for the Alere Determine and Alere Combo tests [15]. However, they were lower than those announced by the manufacturers which ranged from 99.4% to 99.9%. RDTs specificities obtained in this study were also lower than those reported in France for Alere Combo and Vikia tests [16]; but higher than the 94.0% specificity found in Brazil for Alere Determine test [20]. The false-positive results observed with RDTs could be explained by serological cross-reactions due to exposure to various endemic infections (malaria, schistosomiasis, human African trypanosomiasis, etc.), technical problems such as contamination, poor storage of RDT kits, non-compliance with the manufacturer's protocol and misinterpretation of results which can lead to misdiagnosis [21].

This study suggests that a small proportion of patients diagnosed according to the national HIV testing algorithm could have been inappropriately admitted to ARV treatment with consequences such as psychological distress, and long-term liver and kidney damage due to ARVs toxicity in the latter. To ensure the reliability of the results obtained with the RDTs, it would be important not only to test a second time the patients at least three weeks after the first serology but also to confirm their results either by western blot or PCR before putting them under ARVs, contrary to the WHO recommendation “test-and-treat“ consisting of putting all HIV-positive patients under treatment.

**Limitations of the study:** our study has some limitations such as the small size of the sample studied. It concerned only one specific region of Gabon. However, HIV subtypes may vary across the country. We were only able to evaluate three RDTs among many others used in HIV screening in Gabon.

## Conclusion

Despite the high viral genetic diversity of HIV, the sensitivities of the 3 RDTs (100%) obtained in this study complied with WHO recommendations, while their specificities ranged from 94.6% to 96.4%. These specificities would not allow the 3 RDTs to be included in an HIV screening algorithm according to WHO recommendations.

### What is known about this topic

- *Rapid diagnostic tests are widely used in HIV diagnosis in Gabon;*
- *The genetic diversity of HIV can lead to the non-detection of some HIV subtypes*.

### What this study adds

- *The three rapid diagnostic tests (RDTs) showed identical reactivity irrespective of the viral genotypes present;*
- *The specificities were different and lower than those recommended by WHO for inclusion in a diagnostic algorithm;*
- *All three RDTs showed excellent concordance in the detection of HIV-1*.

## References

[1] Direction Générale de la Statistique (DGS) du Gabon et ICF International (2012). 2012 Enquête Démographique et de Santé du Gabon 2012: Rapport de Synthèse.

[2] VIH/sida P, OMS (2016). Dépistage du VIH: recommandations de l'OMS pour garantir la qualité des tests de dépistage du VIH.

[3] Hemelaar J (2012). The origin and diversity of the HIV-1 pandemic. Trends Mol Med.

[4] Yebra G, de Mulder M, Holguin A (2013). Description of HIV-1 group M molecular epidemiology and drug resistance prevalence in Equatorial Guinea from migrants in Spain. PLoS One.

[5] Yamaguchi J, Vallari A, McArthur C, Sthreshley L, Cloherty GA, Berg MG (2020). Complete Genome Sequence of CG-0018a-01 Establishes HIV-1 Subtype L. J Acquir Immune Defic Syndr.

[6] Mouinga-Ondeme A, Mabika-Mabika A, Alalade P, Mongo AD, Sica J, Liegeois F (2014). Significant impact of non-B HIV-1 variants genetic diversity in Gabon on plasma HIV-1 RNA quantitation. J Med Virol.

[7] Ndjoyi-Mbiguino A, Belec L (2005). [Evaluation of a panel of commercial kits for the detection of serum HIV-specific antibodies, and choice of alternative strategies for the serological diagnosis in Libreville (Gabon)]. Sante.

[8] Mintsa-Ndong A, Caron M, Plantier JC, Makuwa M, Le Hello S, Courgnaud V (2009). High HIV Type 1 prevalence and wide genetic diversity with dominance of recombinant strains but low level of antiretroviral drug-resistance mutations in untreated patients in northeast Gabon, Central Africa. AIDS Res Hum Retroviruses.

[9] Engone-Ondo JD, Mouinga-Ondeme A, Lekana-Douki SE, Diane A, Mamimandjiami AI, Banga O (2021). High rate of virological failure and HIV drug resistance in semi-rural Gabon and implications for dolutegravir-based regimen efficacy. J Antimicrob Chemother.

[10] Liegeois F, Boue V, Butel C, Mouinga-Ondeme A, Sica J, Zamba C (2013). HIV type-1 group O infection in Gabon: low prevalence rate but circulation of genetically diverse and drug-resistant HIV type-1 group O strains. AIDS Res Hum Retroviruses.

[11] Ndjoyi-Mbiguino A, Nzengui Nzengui GF, Robin L, M'Boyis Kamdem H, Belec L (2015). Performance of rapid HIV-1/HIV-2 INSTI on plasma and capillary blood for serological diagnosis of non B subtypes and circulating recombinant forms of HIV-1 in Gabon. Med Mal Infect.

[12] Eko Mba JM, Bisseye C, Mombo LE, Ntsame Ndong JM, Mbina Ekayeng SC, Bengone C (2019). Assessment of rapid diagnostic tests and fourth-generation Enzyme-Linked Immuno sorbent Assays in the screening of Human Immunodeficiency and Hepatitis B virus infections among first-time blood donors in Libreville (Gabon). J Clin Lab Anal.

[13] Plantier JC, Djemai M, Lemee V, Reggiani A, Leoz M, Burc L (2009). Census and analysis of persistent false-negative results in serological diagnosis of human immunodeficiency virus type 1 group O infections. J Clin Microbiol.

[14] Vallari A, Holzmayer V, Harris B, Yamaguchi J, Ngansop C, Makamche F (2011). Confirmation of putative HIV-1 group P in Cameroon. J Virol.

[15] Njouom R, Ngono L, Mekinda-Gometi DD, Nde CK, Sadeuh-Mba SA, Vernet MA (2017). Evaluation of the performances of twelve rapid diagnostic tests for diagnosis of HIV infection in Yaounde, Cameroon. J Virol Methods.

[16] Mourez T, Lemée V, Delbos V, Delaugerre C, Alessandri-Gradt E, Etienne M (2018). HIV rapid screening tests and self-tests: Be aware of differences in performance and cautious of vendors. EBioMedicine.

[17] Kashosi TM, Kyambikwa CB, Mulongo PM, Nachega JB (2018). Field accuracy of HIV rapid diagnostic tests for blood donors screening, Bukavu, Eastern Democratic Republic of the Congo. J Infect Dev Ctries.

[18] Beaulieu Q, Jean-Charles P, Costes M, Guilleminault E, Hermet L, Kalkias L (2017). Analyse de la sensibilité de 11 TROD au cours de la primo-infection par du VIH-1. Médecine et Maladies Infectieuses.

[19] Bisseye C, Mombo LE, Bie SMM, Edou A, Eko-Mba JM, Etho-Mengue JC (2018). Trends of blood-borne infectious diseases in a rural blood donation center of southeast Gabon (Koula-Moutou). Pan Afr Med J.

[20] Marinho FLO, Santos NLL, Neves SPF, Vasconcellos LS (2020). Performance evaluation of eight rapid tests to detect HIV infection: A comparative study from Brazil. PLoS One.

[21] Klarkowski D, O'Brien DP, Shanks L, Singh KP (2014). Causes of false-positive HIV rapid diagnostic test results. Expert Rev Anti Infect Ther.

